# Synthesis of calix[4]azacrown substituted sulphonamides with antioxidant, acetylcholinesterase, butyrylcholinesterase, tyrosinase and carbonic anhydrase inhibitory action

**DOI:** 10.1080/14756366.2020.1765166

**Published:** 2020-05-13

**Authors:** Mehmet Oguz, Erbay Kalay, Suleyman Akocak, Alessio Nocentini, Nebih Lolak, Mehmet Boga, Mustafa Yilmaz, Claudiu T. Supuran

**Affiliations:** a Department of Chemistry, University of Selcuk, Konya, Turkey; b Department of Advanced Material and Nanotechnology, Selcuk University, Konya, Turkey; c Kars Vocational School, Kafkas University, Kars, Turkey; d Department of Pharmaceutical Chemistry, Faculty of Pharmacy, Adiyaman University, Adiyaman, Turkey; e NEUROFARBA Department, Sezione di Scienze Farmaceutiche, Università degli Studi di Firenze, Florence, Italy; f Department of Analytical Chemistry, Faculty of Pharmacy, Dicle University, Diyarbakir, Turkey

**Keywords:** Calix[4]azacrown, enzyme inhibition, antioxidant, carbonic anhydrase

## Abstract

A series of novel calix[4]azacrown substituted sulphonamide Schiff bases was synthesised by the reaction of calix[4]azacrown aldehydes with different substituted primary and secondary sulphonamides. The obtained novel compounds were investigated as inhibitors of six human (h) isoforms of carbonic anhydrases (CA, EC 4.2.1.1). Their antioxidant profile was assayed by various bioanalytical methods. The calix[4]azacrown substituted sulphonamide Schiff bases were also investigated as inhibitors of acetylcholinesterase (AChE), butyrylcholinesterase (BChE) and tyrosinase enzymes, associated with several diseases such as Alzheimer, Parkinson, and pigmentation disorders. The new sulphonamides showed low to moderate inhibition against hCAs, AChE, BChE, and tyrosinase enzymes. However, some of them possessed relevant antioxidant activity, comparable with standard antioxidants used in the study.

## 1. Introduction

Carbonic anhydrases are metalloenzymes present in Archaea, prokaryotes, and eukaryotes, and catalyse a physiologically very simple but relevant reaction, i.e. the interconversion of CO_2_ to HCO_3_^−^ and protons via a ping-pong mechanism^1–6^. In humans, 16 different isoforms have been described from eight genetically distinct CA families (α-, β-, γ-, δ-, ζ-, η-, θ-, and ι-CAs)^1–6^. The isozymes have different subcellular localisation, catalytic activity, and inhibitory properties in body fluid and tissues. Since these isoforms play an important role in acid–base regulation, gluconeogenesis and other biosynthetic reactions, electrolyte secretion, bone resorption/calcification, and tumorigenicity, their inhibition/activation may be exploited in several diseases, including glaucoma, obesity, neuropathic pain, arthritis, Alzheimers` disease and more recently cancer^1–6^.

Acetylcholinesterase (AChE; EC 3.1.1.7), which hydrolyses the neurotransmitter acetylcholine, is found in high concentrations over the peripheral and central nervous systems but also in other tissues^7^^,^^8^. The imbalance in AChE activity can cause various types of neurodegenerative pathologies such as Alzheimer’s disease (AD) and Parkinson’s disease (PD). Butyrylcholinesterase (BChE) is a non-specific cholinesterase enzyme that hydrolyses many different choline-based esters^9^^,^^10^. Thus, AChE and BChE inhibition have been documented as an interesting approach in the palliative treatment of AD^11^^,^^12^.

Several major classes of macrocyclic derivatives such as the crown ethers, cyclodextrins, and calixarenes are important third-generation derivatives in supramolecular chemistry^13^^,^^14^. Among them, calixarenes may have biomedical applications thanks to their exceptional structural properties, which may be exploited for designing antitumor, antiviral, antimicrobial, anti-thrombotic, and antifungal derivatives^15–17^. From this perspective, synthesising new derivatised calixarenes as antioxidant and enzyme inhibitors, and exploring the molecular mechanisms underlying their effect are ongoing new fields.

In continuation of our recent interest in developing new metalloenzymes inhibitors^18–21^, in this study, we report the first calix[4]azacrown substituted sulphonamide Schiff bases (to the best of our knowledge) acting as an antioxidant and metalloenzyme inhibitors (such as carbonic anhydrase, acetylcholinesterase, butyrylcholinesterase, and tyrosinase inhibitors).

## 2. Materials and methods

### 2.1. General

All materials were purchased from commercial suppliers and used without further purification. All reactions were conducted under an atmosphere of nitrogen unless noted otherwise. Anhydrous solvents were distilled over appropriate drying agents before use. ^1^H NMR spectra were recorded on a Varian 400 MHz spectrometer in DMSO-d_6_ solution with the internal solvent signal peak at 2.50 ppm. 13C NMR was recorded at 100 MHz spectrometer in DMSO-d_6_ solution and referenced to the internal solvent signal at 39.5 ppm. Proton NMR data are reported as follows: chemical shift (ppm), multiplicity (s = singlet, d = doublet, t = triplet, q = quartette, dd = doublet of doublets, dt = triplet of doublets, m = multiplet, brs. = broad singlet), and coupling constants (Hz). Infra-red spectra were measured using a Bruker Spectrometer transform-infra-red (FT-IR) spectrometer. The progress of the reactions was monitored by thin layer chromatography. For the detection of the spots, the plates were exposed to UV light (254 and 365 nm).

### 2.2. Synthesis procedure for preparation of sulphonamides

#### 2.2.1. Synthesis of SA-1


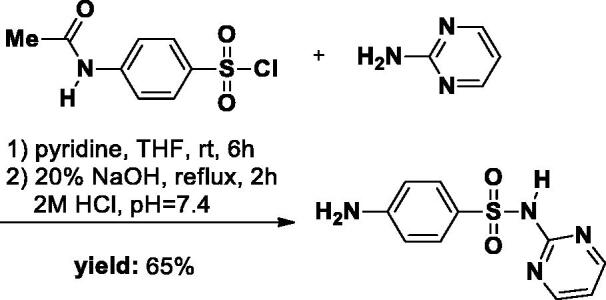


The **SA-1** was synthesised as described in literature^22^. Briefly, pyrimidin-2-amine (1.14 g, 12 mmol) was added to a 50 ml round-bottom flask and dissolved in a solution of THF (20 ml) and pyridine (1 ml). *p*-Acetamidobenzenesulfonyl chloride (2.81 g, 12 mmol) (6 g, 2.81 mmol) was then added to the reaction solution. After completion of the reaction followed by TLC, the THF was removed in the evaporator. Subsequently, 30 ml 0.5 M HCl was added and a large amount of precipitate was formed. The precipitate was filtrated and then washed with water and used directly in the next step. The Intermediate was dissolved in a 20% NaOH solution and the reaction mixture was refluxed for 2 h. After the reaction was completed, the mixture was quenched to approximately pH 7.4 with 2 M HCl, resulting in a large number of white solids. The precipitate was obtained and washed with water, then dried overnight in a desiccator (white solid, yield 65%).

#### 2.2.2. Synthesis of SA-2


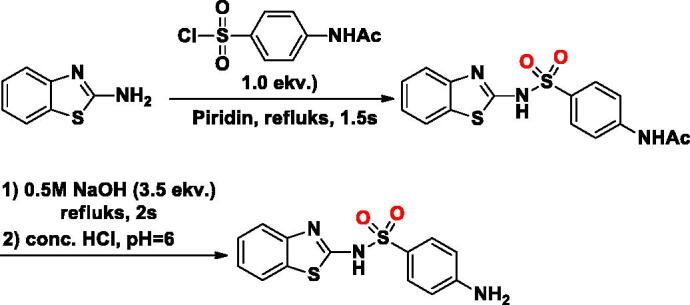


The **SA-2** was synthesised as described in literature^23^. Briefly, in a 50 ml round-bottom flask, to a stirred solution of benzo[*d*]thiazol-2-amine (1.0 g, 6.66 mmol) in dry pyridine (6 ml) was added 4-acetamidobenzenesulfonyl chloride (1.55 g, 6.66 mmol). The reaction mixture was refluksed for 2 h. The reaction mixture was poured onto acidic crushed ice and filtered. The crude solid was washed with water and directly used in the next step. The intermediate was dissolved in 1 M NaOH solution (3.5 ekv. of the intermediate) and the reaction mixture was refluksed for 2 h before being cooled down to room temperature. Then the reaction solution was quenched with 2 M HCl to approximately pH 6, resulting in a large number of white solids. To obtain the desired product, the crude product was filtered, washed with water, and then dried in the desiccator (white solid, yield 68%).

#### 2.2.3. Synthesis of SA-3


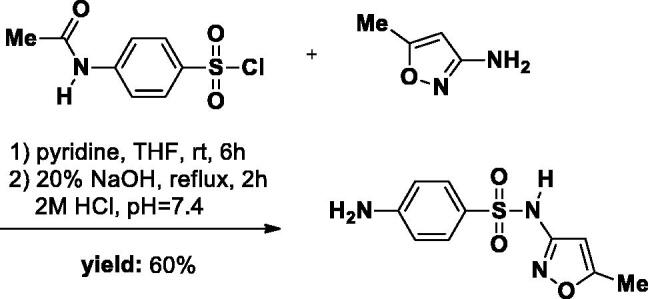


The **SA-3** was synthesised as described in literature^24^. Briefly, 3-amino-5-methylisoxazole (1.18 g, 12 mmol) was added to a 50 ml round-bottom flask and dissolved in a solution of THF (20 ml) and pyridine (1 ml). *p*-Acetamidobenzensulfonyl chloride (6 g, 2.81 mmol) was then added to the reaction solution. The reaction mixture was stirred at room temperature for 12 h. The reaction was followed by TLC. After completion of the reaction, the THF was removed in the evaporator. Subsequently, 30 ml 0.5 M HCl was added to the crude mixture and a large amount of precipitate was formed. The precipitate was filtrated, washed with water, and directly used in the next step. The Intermediate was dissolved in a 20% NaOH solution and the reaction mixture was refluxed for 2 h. After the reaction was completed, the mixture was quenched to approximately pH 7.4 with 2 M HCl, resulting in a large number of white solids. The precipitate was obtained and washed with water, then dried overnight in a desiccator (white solid, yield 60%).

#### 2.2.4. Synthesis of SA-4 and SA-5


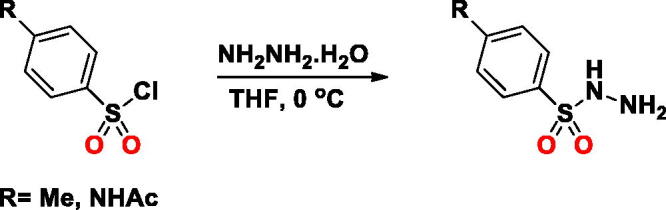


***General Procedure:*** The **SA-5** and **SA-6** were synthesised as described in literature^25^. Briefly, a solution of an arylsulfonyl chloride (10 mmol) in tetrahydrofuran (40 ml) was added hydrazine monohydrate (1.25 ml, 2.5 equiv.) dropwise at 0 °C. The reaction mixture was stirred vigorously for 30 min at 0 °C. Upon completion, the reaction mixture was added ethyl acetate (50 ml) and extracted with saturated brine (3 × 50 ml). The organic layer was separated, dried over anhydrous Na_2_SO_4_, filtrated, concentrated in an evaporator and added to hexane (10 ml) over 5 min. The precipitate was filtered, collected, and dried in vacuum.

### 2.3. General procedure for the synthesis of compounds CX (1–6)

In a 25-ml round-bottomed flask equipped with a magnetic stirrer, sulphonamide derivative (0.4 mmol) was added to the solution of calix[4]arene-aldehyde (0.2 mmol, 146.6 mg) in a mixture of 10 ml CHCl_3_/MeOH (1:1). The resulting mixture heated to reflux overnight. After the reaction was complete, the solvent was removed. The crude mixture was dissolved with 2 ml of methylene chloride. Upon the addition of hexane to the solution, the target product was precipitated. Then, the product was filtered off and dried under vacuum at 40 °C. The obtained final pure compounds CX(1–6) were fully characterised by ^1^H-NMR and ^13^C-NMR techniques.

**CX-1:** A white solid, yield 717%. ^1^H NMR (400 MHz, DMSO-d_6_) δ; 9.19 (s, 2H, CONH), 8.34 (s, 2H, CH = N), 7.88 (d, *J* = 8.5 Hz, 4H, ArH) , 7.57 (d, *J* = 8.5 Hz, 4H, ArH) , 7.55 (s, 4H, calix-ArH), 7.11 (s, 4H, calix-ArH), 4.51 (s, 4H, OCH_2_), 4.20 (d, *J* = 13.5 Hz, 4H, ArCH_2_Ar), 3.69 (d, *J* = 13.5 Hz, 4H, ArCH_2_Ar), 3.49 (m, 4H, CH_2_CH_2_), 1.10 (s, 18H, *^t^*Bu); ^13^C NMR (100 MHz, DMSO-d_6_) δ 166.9, 161.4, 153.2, 150.0, 141.9, 140.3, 131.7, 130.3, 129.6, 129.0, 128.3, 127.6, 126.8, 125.8, 74.1, 43.6, 38.6, 34.4, 32.5; Anal. Calcd for C_64_H_66_N_8_O_12_S_2_ (1041.24): C, 64.60; H, 5.81; N, 8.07; S, 6.16. Found: C, 64.83; H, 5.80; N, 8.07; S, 6.14.

**CX-2:** A yellow solid, yield 70%. ^1^H NMR (400 MHz, DMSO-d_6_) δ 9.31 (s, 2H, CONH), 8.30 (s, 2H, CH = N), 7.92 (d, *J* = 8.5 Hz, 4H, ArH), 7.60 (d, *J* = 8.5 Hz, 4H, ArH), 7.56 (s, 4H, calix-ArH), 7.15 (s, 4H, calix-ArH), 6.07 (s, 2H, Het-ArH), 4.50 (s, 4H, OCH_2_), 4.21 (d, *J* = 13.5 Hz, 4H, ArCH_2_Ar), 3.68 (d, *J* = 13.5 Hz, 4H, ArCH_2_Ar), 3.50 (m, 4H, CH_2_CH_2_), 2.25 (s, 6H, Me), 1.12 (s, 18H, *^t^*Bu); ^13^C NMR (100 MHz, DMSO-d_6_) δ 168.8, 167.7, 160.5, 152.2, 151.1, 150.4, 143.3, 141.9, 132.4, 130.1,129.7, 129.2, 128.7, 127.1, 126.3, 124.6, 95.6, 74.7, 42.0, 38.7, 34.4, 32.0, 12.9**;** Anal. Calcd for C_64_H_66_N_8_O_12_S_2_ (1197.38): C, 63.88; H, 5.53; N, 9.31; S, 5.33. Found: C, 63.97; H, 5.50; N, 9.29; S, 5.35.

**CX-3**: A yellow solid, yield 72%. ^1^H NMR (400 MHz, DMSO-d_6_) δ 9.42 (s, 2H, CONH), 8.45 (d, *J* = 8.8 Hz, 4H, Het-ArH), 8.33 (s, 2H, CH = N), 7.96 (d, *J* = 8.8 Hz, 4H, ArH), 7.60 (d, *J* = 8.8 Hz, 4H, ArH), 7.58 (s, 4H, calix-ArH), 7.15 (s, 4H, calix-ArH), 6.97 (t, *J* = 4.8 Hz, 2H, ArH), 4.50 (s, 4H, OCH_2_), 4.21 (d, *J* = 13.4 Hz, 4H, ArCH_2_Ar), 3.70 (d, *J* = 13.4 Hz, 4H, ArCH_2_Ar), 3.50 (m, 4H, CH_2_CH_2_), 1.12 (s, 18H, *^t^*Bu); ^13^C NMR (100 MHz, DMSO-d_6_) δ 167.4, 162.6, 159.7, 158.0, 157.4, 157.1, 155.9, 144.5, 142.2, 138.8,130.1, 129.9, 129.2, 128.7, 128.3, 127.7, 126.0, 75.6, 44.0, 38.2, 34.2, 31.1; Anal. Calcd for C_64_H_64_N_10_O_10_S_2_ (1197.38): C, 64.20; H, 5.39; N, 11.70; S, 5.36. Found: C, 64.42; H, 5.41; N, 11.74; S, 5.37.

**CX-4:** A yellow solid, yield 70%. ^1^H NMR (400 MHz, DMSO-d_6_) δ 9.41 (bs, 2H, CONH), 8.31 (s, 2H, CH = N), 7.92 (d, *J* = 8.5 Hz, 4H, ArH), 7.85–7.70 (m, 4H, Het-ArH), 7.57 (s, 4H, calix-ArH), 7.55–7.48 (m, 8H, ArH), 7.14 (s, 4H, calix-ArH), 4.50 (s, 4H, OCH_2_) 4.21 (d, *J* = 13.5 Hz, 4H, ArCH_2_Ar), 3.69 (d, *J* = 13.5 Hz, 4H, ArCH_2_Ar), 3.49 (m, 4H, CH_2_CH_2_), 1.12 (s, 18H, *^t^*Bu); ^13^C NMR (100 MHz, DMSO-d_6_) δ 169.6, 166.1, 161.9, 152.6, 152.0, 151.4, 147.3, 144.2, 132.5, 130.9, 130.0, 129.4, 128.7, 128.2, 127.9, 127,4, 127.4, 127.0, 126.8, 126.2, 125.4, 75.9, 44.0, 38.5, 34.6, 31.0; Anal. Calcd for C_70_H_66_N_8_O_10_S_4_ (1307.58): C, 64.30; H, 5.09; N, 8.57; S, 9.81. Found: C, 64.49; H, 5.09; N, 8.59; S, 9.79.

**CX-5:** As a yellow solid, yield 90%. ^1^H NMR (400 MHz, DMSO-d_6_) δ 8.92 (s, 2H, CONH), 8.31 (s, 2H, CH = N), 7.72 (d, *J* = 8.5 Hz, 4H, ArH), 7.45 (s, 4H, calix-ArH), 7.34 (d, *J* = 8.5 Hz, 4H, ArH), 7.15 (s, 4H, calix-ArH), 2.30 (s, 6H, Me), 4.46 (s, 4H, OCH_2_), 4.10 (d, *J* = 13.2 Hz, 4H, ArCH_2_Ar), 3.58 (d, *J* = 13.2 Hz, 4H, ArCH_2_Ar), 3.47 (m, 4H, CH_2_CH_2_), 2.30 (s, 6H, Me), 1.09 (s, 18H, *^t^*Bu); ^13^C NMR (100 MHz, DMSO-d_6_) δ 167.6, 153.1, 151.9, 148.2, 143.7, 141.4, 133.6, 131.8, 130.0, 129.5, 129.1, 128.2, 127.3, 125.6, 75.5, 44.3, 38.2, 34.5, 30.9, 21.8; Anal. Calcd for C_58_H_64_N_6_O_10_S_2_ (1069.29): C, 65.15; H, 6.03; N, 7.86; S, 6.00. Found: C, 65.39; H, 6.02; N, 7.86; S, 5.59.

**CX-6:** As a yellow solid. (203 mg, yield 88%); ^1^H NMR (400 MHz, DMSO-d_6_) δ 8.89 (s, 2H, CONH), 8.30 (s, 2H, CH = N), 7.78 (d, *J* = 9.0 Hz, 4H, ArH), 7.73 (d, *J* = 9.0 Hz, 4H, ArH), 7.42 (s, 4H, calix-ArH), 7.11 (s, 4H, calix-ArH), 2.30 (s, 6H, Me), 4.46 (s, 4H, OCH_2_), 4.10 (d, *J* = 13.1 Hz, 4H, ArCH_2_Ar), 3.57 (d, *J* = 13.1 Hz, 4H, ArCH_2_Ar), 3.47 (m, 4H, CH_2_CH_2_), 2.03 (s, 6H, Me), 1.06 (s, 18H, *^t^*Bu); ^13^C NMR (100 MHz, DMSO-d_6_) δ 168.2, 167.6, 156.9, 152.4, 147.9, 143.0, 141.2, 132.2, 131.0, 129.7, 129.2, 128.4, 127.5, 126.4, 124.9, 75.2, 44.1, 38.0, 34.0, 30.1, 23.8; Anal. Calcd for C_60_H_66_N_8_O_12_S_2_ (1155.34): C, 62.37; H, 5.76; N, 9.70; S, 5.55. Found: C, 62.59; H, 5.76; N, 9.73; S, 5.54.

### 2.4. CA inhibition assay

An SX.18 MV-R Applied Photophysics (Oxford, UK) stopped-flow instrument has been used to assay the inhibition of various CA isozymes^26^. Phenol Red (at a concentration of 0.2 mM) has been used as an indicator, working at the absorbance maximum of 557 nm, with 10 mM Hepes (pH 7.4) as a buffer, 0.1 M Na_2_SO_4_ or NaClO_4_ (for maintaining constant the ionic strength; these anions are not inhibitory in the used concentration), following the CA-catalyzed CO_2_ hydration reaction for a period of 5–10 s. Saturated CO_2_ solutions in water at 25 °C were used as substrate. Stock solutions of inhibitors were prepared at a concentration of 10 mM (in DMSO-water 1:1, v/v) and dilutions up to 0.01 nM done with the assay buffer mentioned above. At least 7 different inhibitor concentrations have been used for measuring the inhibition constant. Inhibitor and enzyme solutions were pre-incubated together for 10 min at room temperature before assay, to allow for the formation of the E-I complex. Triplicate experiments were done for each inhibitor concentration, and the value reported throughout the paper is the mean of such results. The inhibition constants were obtained by nonlinear least-squares methods using the Cheng-Prusoff equation, as reported earlier, and represent the mean from at least three different determinations1^27–32^. All CA isozymes used here were recombinant proteins obtained as reported earlier by our group.

### 2.5. Determination of antioxidant, anticholinesterase and tyrosinase activity of calix[4]arene sulphonamides CX(1–6)

#### 2.5.1. Dpph radical scavenging assay

The DPPH (2,2-diphenyl-1-picrylhydrazyl) radical scavenging activity of the synthesised compounds was determined by a spectrophotometric method based on the reduction of an ethanol solution of DPPH^33^^,^^34^. 2, 5, 10, 20 µL of 1 mM stock solution of each compound was completed to 40 µL with the DMSO and mixed with 160 µL of 0.1 mM of DPPH free radical solution. The mixture was led to stand for 30 min in the dark and the absorbance was then measured at 517 nm against a blank. Inhibition of free radical, DPPH, in percent (I %), was calculated according to the formula:

$$I\;\%=\left(A_{\text{control}}-A_{\text{sample}}\right)/A_{\text{control}}\times 100$$

where A_control_ is the absorbance of the control reaction (containing all reagents except for the tested compounds), and A_sample_ is the absorbance of the test compounds. Tests were carried out in triplicate. BHA and BHT were used as positive control.

#### 2.5.2. Abts cation radical decolorisation assay

The percent inhibition of decolorisation of ABTS [2,2′-azino-bis(3-ethylbenzothiazoline-6-sulfonic acid)] cation radical is obtained as a function of time and concentration and evaluated by comparison with the BHT and BHA compounds used as standard^35^^,^^36^. The tested compounds at different concentrations are added to each well and 160 µL of 7 mM ABTS solution is added. After 6 min at room temperature, the absorbances were measured at 734 nm. ABTS cation radical decolorisation activities were determined by using the equation below:

$$\%\text{Inhibition}\;=\;\left({\text{A}}_{\text{control}}-{\text{A}}_{\text{sample}}\right)/{\text{A}}_{\text{control}}\;\times \;\text{1}00$$

where A is the absorbance. Tests were carried out in triplicate. BHA and BHT were used as positive control.

#### 2.5.3. Metal chelating activity

The chelating ability of synthesised compounds was examined according to the method of Dinis et al.^37^. The tested compounds at different concentrations were added to each well and 4 µL of 2 mM ferrous (II) chloride was added. Then 8 µL of 5 mM ferrozine was added and the reaction was started. After 10 min at room temperature, the absorbance was measured at 562 nm against a blank. The results were expressed as a percentage of inhibition of the ferrozine-Fe^2+^ complex formation. The percentage inhibition of the ferrozine -Fe^2+^ complex formation was calculated using the formula given below:

$$\text{Chelating}\;\text{ability}\;\left(\%\right)=\left(A_{\text{control}}-A_{\text{sample}}\right)/A_{\text{control}}\times 100$$

where A is the absorbance. Tests were carried out in triplicate. EDTA was used as a positive control.

#### 2.5.4. Anticholinesterase assay

The inhibitory effect of novel calix[4]azacrown substituted sulphonamide Schiff bases **CX(1–6)** on AChE and BChE activities was determined according to the slightly modified spectrophotometric method of Ellman et al.^38^. All compounds were dissolved in DMSO to prepare stock solutions at 4 mM concentration. Aliquots of 150 µL of 100 mM sodium phosphate buffer (pH 8.0), 10 µL of sample solution and 20 µL AChE (or BChE) solution were mixed and incubated for 15 min at 25 °C, and DTNB [5,5′-Dithio-bis(2-nitro-benzoic)acid] (10 µL) is added. The reaction was then initiated by the addition of acetylthiocholine iodide (or butyrylthiocholine iodide) (10 µL). The final concentration of the tested compounds’ solution was 200 µM.

$$\%\text{Inhibition}=\left(A_{\text{control}}-A_{\text{sample}}\right)/A_{\text{control}}\times 100$$

where A is the absorbance. Tests were carried out in triplicate. Galantamine was used as positive control.

#### 2.5.5. Anti-tyrosinase activity

Anti-tyrosinase activity of the compounds was performed according to the method designed by Hearing and Jimenez^39^. Firstly, the inhibition of diphenolase function of the compounds was evaluated and L-DOPA was used as substrate. Tyrosinase from mushroom (E.C. 1.14.18.1) (30 U, 28 nM) was dissolved in Na-phosphate buffer (pH = 6.8, 50 nM) and the compounds were added to the solution for pre-incubation at room temperature for ten minutes. After incubation, 0.5 mM L-DOPA was added to the mixture and the change in absorbance was measured at 475 nm at 37 °C. For positive control, kojic acid was used. The following formula was used to calculate the percentage of all enzyme inhibitions:

$$\text{Inhibition }\left(\%\right)\;=\;\left({\text{A}}_{\text{control}}–{\text{A}}_{\text{sample}}\right)/{\text{A}}_{\text{control}}\;\times \;\text{1}00$$

A: Absorbance.

### 2.6. Statistical analysis

The results of the antioxidant, anticholinesterase, and tyrosinase activity assays are expressed as the mean ± SD of three parallel measurements. The statistical significance was estimated using a Student’s *t*-test, where *p* values < 0.05 were considered significant.

## 3. Results and discussion

### 3.1. Chemistry

**Scheme 1.**
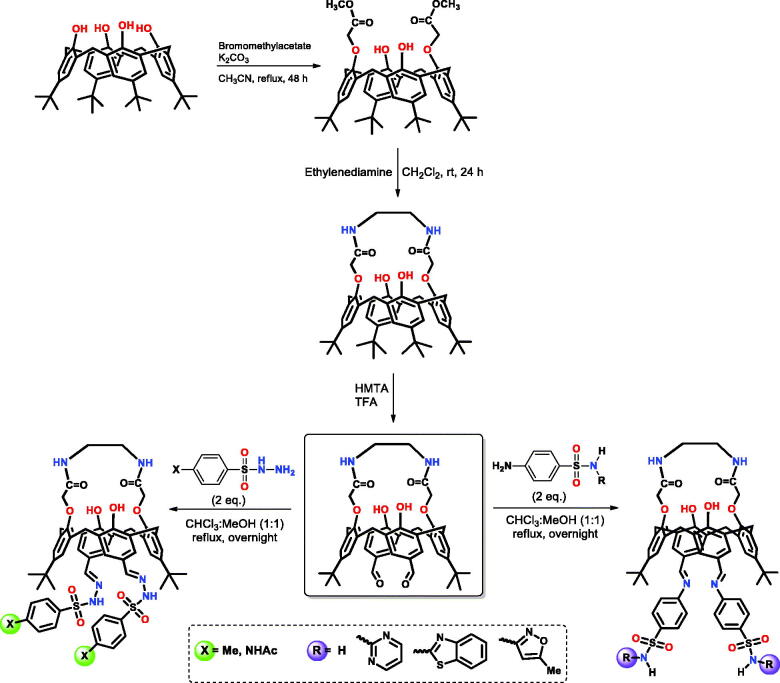
General synthetic route for the synthesis of the calix[4]arene substituted sulphonamide Schiff base derivatives **CX(1–6)**.

To develop novel and effective enzyme inhibitors and antioxidant agents based on calixarenes, we used the calix[4](aza)crown dialdehyde as a scaffold to design a series of new derivatives bearing different sulphonamide moieties. The sulphonamide-substituted calix[4]zacrown derivatives **CX(1–6)** were obtained in four steps (Scheme 1). The required starting compound *p-tert*-butylcalix[4]arene was synthesised according to literature procedure^16^ and then the calix[4]arene diester was obtained in satisfactory yield by reflux with bromomethyl acetate in the presence of K_2_CO_3_ in CH_3_CN^16^. The diester derivative of calixarene was reacted with ethylenediamine in CH_3_OH/CHCl_3_ (1:1) at room temperature, being converted to *p-tert-*butycalix[4](aza-)crown derivative which was isolated by recrystallisation from the methanol^16^. The desired *p-tert*-butycalix[4](aza)crown dialdehyde derivative was prepared by treating *p-tert*-butycalix[4](aza)crown with hexamethylenetetraamine with refluxing trifluoroacetic acid^16^. In the last step, the sulphonamide derivatives (**SA-1–5)** were reacted with calix(aza)crown dialdehyde in absolute ethanol to synthesise the novel calix(aza)crown substituted sulphonamide derivatives **CX(1–6)**. All the synthesised compounds were fully characterised by using spectroscopic techniques (see the experimental part for details).

### 3.2. CA inhibition studies

The newly synthesised calix[4]azocrown substituted sulphonamide Schiff bases C**X(1–6)** were assessed as inhibitors of six physiologically relevant CA isoforms, the cytosolic hCA I, hCA II, and hCA VII, membrane-bound hCA IV, and the transmembrane tumor-associated hCA IX and hCA XII, by a stopped flow CO_2_ hydrase assay^26^. The clinically used sulphonamide acetazolamide (**AAZ**) was used as a positive control. The compounds, in general, showed low inhibition potency against the tested CA isoforms. Specifically, all the secondary sulphonamides exhibited inhibition constants >100 µM activity against the tested CA isoforms, except the isoform hCA IX, for which some of the compounds (**CX-2**, **CX-3** and **CX-6**) showed moderate activity with K_I_s of 67.6, 46.0, and 64.6 µM, respectively. On the other hand, the primary sulphonamide substituted derivative (**CX-1**) showed medium potency activity against the tumor-associated isoforms hCA IX and hCA XII with K_I_s in the range of 0.15 to 0.27 µM, respectively, also inhibiting in the micromolar ramge the other isoforms (Table 1).

**Table 1.** *In vitro* hCA I, hCA II, hCA IV, hCA VII, hCA IX, and hCA XII inhibition data with calix[4]azacrown substituted sulphonamide Schiff base derivatives **CX(1–6)** investigated here, and standard sulphonamide inhibitor Acetazolamide (**AAZ**) by a stopped flow CO_2_ hydrase assay^26^.

	K_I_^a^ (µM)					
Compound	hCA I	hCA II	hCA IV	hCA VII	hCA IX	hCA XII
**CX-1**	5.55	0.82	4.36	1.21	0.15	0.27
**CX-2**	>100	>100	>100	>100	67.6	>100
**CX-3**	>100	>100	>100	>100	46.0	10.2
**CX-4**	>100	>100	>100	>100	>100	>100
**CX-5**	>100	>100	>100	>100	>100	>100
**CX-6**	>100	>100	>100	>100	64.6	>100
**AAZ**	0.25	0.01	0.07	0.002	0.02	0.006

^a^Mean from 3 different assays, by a stopped flow technique (errors were in the range of ± 5–10% of the reported values).

### 3.3. Antioxidant activity

The antioxidant capacities of the newly synthesised compounds **CX(1–6)** were demonstrated by using three different methods, namely, DPPH free radical scavenging, ABTS cation radical scavenging, and metal chelating methods. All of the compounds showed antioxidant activities in a dose-dependent manner and shown in Table 2, and the IC_50_ values were compared with the standards BHA, BHT, and EDTA. The three compounds (**CX-1**, **CX-2,** and **CX-3**) showed no activity against DPPH free radical assay with IC_50_ values of >1000 μM, but **CX-5** and **CX-6** had an activity comparable with standards, having IC_50_ values of 16.79 ± 0.85 and 9.02 ± 0.05 μM, respectively. Interestingly, these two compounds (**CX-5** and **CX-6**) were also sensitive to ABTS radical scavenging activity with IC_50_ values of 9.79 ± 0.09 and 7.74 ± 0.04 μM, respectively. On the other hand, none of the tested compounds showed any metal chelating activity.

**Table 2.** The antioxidant activity of calix[4]azacrown substituted sulphonamide Schiff base derivatives **CX(1–6)** and controls BHA, BHT, and EDTA.

	IC_50_ values (μM)^a^		
Samples	DPPH Free Radical	ABTS Cation Radical	Metal Chelate
**CX-1**	>1000	769.97 ± 0.22	>1000
**CX-2**	>1000	>1000	>1000
**CX-3**	>1000	121.03 ± 0.95	>1000
**CX-4**	520.33 ± 0.89	>1000	>1000
**CX-5**	16.79 ± 0.85	9.79 ± 0.09	>1000
**CX-6**	9.02 ± 0.05	7.74 ± 0.04	>1000
BHA^b^	7.88 ± 0.20	17.59 ± 0.10	–
BHT^b^	58.86 ± 0.50	13.25 ± 0.27	–
EDTA^b^	–	**–**	26.82 ± 0.10

^a^ IC_50_ values represent the means (standard deviation of three parallel measurements (*p* < 0.05).

^b^ Reference compounds.

### 3.4. Acetylcholinesterase, butyrylcholinesterase, and tyrosinase activity

The calix[4]azacrown substituted sulphonamide Schiff bases **CX(1–6)** were also evaluated for their anti-cholinesterase (AChE and BChE) and anti-tyrosinase activities. None of the compounds from the series showed any inhibition potency against AChE and BChE enzymes, except for compounds **CX-6**, which showed moderate activity against BChE with % inhibition value of 35.41 ± 0.90. The tyrosinase activity of the compounds was also moderate and close the each other, with % inhibition values in the range of 16.48 ± 0.21 to 35.52 ± 0.82, except compound **CX-5**, which showed no activity against tyrosinase (Table 3).

**Table 3.** Anti-cholinesterase and anti-tyrosinase activity of calix[4]azacrown substituted sulphonamide Schiff base derivatives **CX(1–6)** and controls galantamine and kojik acid.

Samples	AChE assay^a^	BChE assay^a^	Tyrosinase activity^a^
**CX-1**	NA	NA	24.46 ± 0.53
**CX-2**	NA	NA	19.55 ± 0.43
**CX-3**	NA	NA	35.52 ± 0.82
**CX-4**	NA	NA	16.48 ± 0.21
**CX-5**	NA	NA	NA
**CX-6**	NA	35.41 ± 0.90	28.15 ± 0.74
Galantamine^b^	80.69 ± 0.59	76.50 ± 1.28	–
Kojik acid^b^	–	–	95.26 ± 0.23

^a^ % inhibition values at 200 µM.

^b^ Standard drugs. NA: not active.

## 4. Conclusion

In the current work, we report a novel series of six calix[4]azacrown substituted sulphonamide Schiff bases which were synthesised by the reaction of calix[4]arene dialdehydes with different substituted primary and secondary sulphonamide derivatives. The newly synthesised novel compounds were investigated as antioxidant and metabolic enzyme inhibitors namely, carbonic anhydrase, acetylcholinesterase, butyrylcholinesterase, and tyrosinase enzymes. The results revealed that these calix[4]azacrown based sulphonamides **CX(1–6)** show, in general, low to moderate metabolic enzyme inhibition against hCAs, AChE, BChE, and tyrosinase enzymes. More specifically, only primary sulphonamide substituted compound (**CX-1**) showed moderate activity against six different isoforms of carbonic anhydrases with K_i_ values ranging from 0.15 to 5.55 µM. On the other hand, some of the synthesised compounds showed great antioxidant activity comparable with standards used in the study, such as **CX-6** (IC_50_ of 9.02 ± 0.05 μM) for DPPH radical scavenging assay and **CX-5** and **CX-6** (IC_50_ 9.79 ± 0.09 and 7.74 ± 0.04 μM, respectively) for ABTS radical decolarisation assay.
